# TDAG8, TRPV1, and ASIC3 involved in establishing hyperalgesic priming in experimental rheumatoid arthritis

**DOI:** 10.1038/s41598-017-09200-6

**Published:** 2017-08-21

**Authors:** Wei-Shan Hsieh, Chia-Chi Kung, Shir-Ly Huang, Shih-Chang Lin, Wei-Hsin Sun

**Affiliations:** 1 Department of Life Sciences, National Central University, Zhongli, Taoyuan city Taiwan; 2 Department of Anesthesiology, National Taiwan University Hospital Hsin-Chu Branch, Hsin-Chu, Taiwan; 3 Institute of Microbiology and Immunology, National Yang-Ming University, Taipei, Taiwan; 4 Department of Immunology, Cathy General Hospital, Taipei, Taiwan

## Abstract

Rheumatoid arthritis (RA), characterized by chronic inflammation of synovial joints, is often associated with ongoing pain and increased pain sensitivity. High hydrogen ion concentration (acidosis) found in synovial fluid in RA patients is associated with disease severity. Acidosis signaling acting on proton-sensing receptors may contribute to inflammation and pain. Previous studies focused on the early phase of arthritis (<5 weeks) and used different arthritis models, so elucidating the roles of different proton-sensing receptors in the chronic phase of arthritis is difficult. We intra-articularly injected complete Freund’s adjuvant into mice once a week for 4 weeks to establish chronic RA pain. Mice with knockout of acid-sensing ion channel 3 (ASIC3) or transient receptor potential/vanilloid receptor subtype 1 (TRPV1) showed attenuated chronic phase (>6 weeks) of RA pain. Mice with T-cell death-associated gene 8 (TDAG8) knockout showed attenuated acute and chronic phases of RA pain. TDAG8 likely participates in the initiation of RA pain, but all three genes, TDAG8, TRPV1, and ASIC3, are essential to establish hyperalgesic priming to regulate the chronic phase of RA pain.

## Introduction

Rheumatoid arthritis (RA) affects approximately 1% of the global population, for one of the most prevalent chronic health problems. RA is an autoimmune disease of the synovium that leads to an inflammatory polyarthritis. Chronic joint inflammation leads to cartilage damage and ultimately total joint destruction^1^. The joint inflammation is often accompanied by ongoing pain and increased pain during movement and light pressure to the articular margin of the joint^2^. In many people, RA may be well controlled, but pain often lingers. Chronic pain with RA becomes independent, essentially becoming its own disease. The assessment of arthritic pain is of critical importance for better understanding the underlying mechanisms of the disease and evaluating therapeutic targets.

High hydrogen ion concentration [H^+^] (acidosis) in synovial fluid is associated with RA disease activity^3^. Local tissue acidosis is a dominant factor in pain and hyperalgesia induced by inflammation^4, 5^. Thus, RA-associated chronic pain is likely triggered by acidosis via proton-sensing receptors. Several proton-sensing receptors are involved in arthritis or arthritis-associated pain. Deletion of acid-sensing ion channel 3 (ASIC3) can reduce secondary mechanical hyperalgesia induced by carrageenan injection or anti-collagen antibody/lipopolysaccharide injection^6, 7^. Although ASIC3 deficiency can reduce arthritic pain, it increases synovial inflammation^7^. ASIC3 is expressed in joint afferents^8^ but also synovial cells and cartilage^9^, which may explain the different outcomes. Use of a selective ASIC3 blocker, APETx2, attenuated disease and pain progression of early-phase osteoarthritis (OA) in a rat model^10^. Thus, ASIC3 deficiency can reduce acute arthritic pain, but whether it increases inflammation in this disease phase is unknown.

In an arthritis model induced by complete Freund’s adjuvant (CFA) injection in C57BL/6 mice, deletion of transient receptor potential/vanilloid receptor subtype 1 (TRPV1) attenuated joint and paw swelling, mechanical hyperalgesia, synovial inflammation, bone erosion, and cartilage damage in the early disease phase (≤5 weeks)^11–13^. In addition to human synovial cells containing ion channels, proton-sensing G-protein-coupled receptors (GPCRs) were reported to respond to protons to cause calcium release^14^, which suggests their involvement in arthritis. Deletion of T-cell death-associated gene 8 (TDAG8), a proton-sensing GPCR, increased the severity of anti-collagen antibody/lipopolysaccharide-induced arthritis, but arthritis-induced pain was not assessed in this study^15^.

All these previous studies focused on the acute phase of arthritis (<5 weeks) and used different arthritis models to study proton-sensing genes, but pain and the roles of proton-sensing receptors in the chronic phase remain unclear. In this study, we established a chronic arthritis model in ICR mice to explore the roles of different proton-sensing receptors in RA-associated pain and inflammation. ICR mice with CFA injection once a week for 4 weeks showed long-term inflammation and bilateral hyperalgesia for 12 weeks. ASIC3 or TRPV1 deficiency attenuated arthritis-associated hyperalgesia in the chronic phase (after 6 or 8 weeks) and TDAG8 knockout attenuated the hyperalgesia in the acute and chronic phases, but TDAG8 knockdown only attenuated acute phase (before 4 weeks) of RA pain. ASIC3 or TRPV1 deficiency suppressed TDAG8, TRPV1 and ASIC3 expression at week 12. TDAG8 knockdown suppressed ASIC3 and TRPV1 gene expression at weeks 4 or 8, respectively. However, it did not suppress expression of these two genes at week 12. The continuous inhibition of TDAG8, TRPV1 and ASIC3 expression in the late phase could be essential to attenuated chronic phase of RA pain. Accordingly, TDAG8, TRPV1, and ASIC3 participate in establishing the chronic phase of RA pain.

## Results

### Arthritic animals show long-lasting inflammation and mechanical hyperalgesia

**Figure 1:**
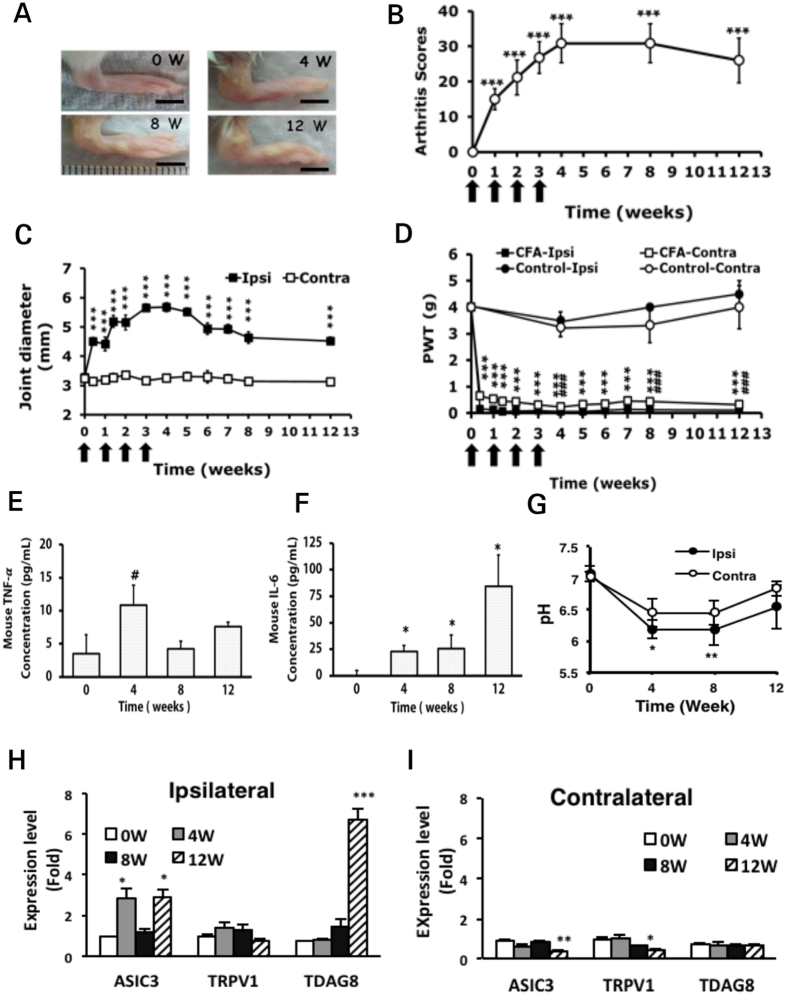
Arthritic ICR mice show long-lasting inflammation and bilateral mechanical hyperalgesia. Mice were injected with 5 μg complete Freund’s adjuvant (CFA) in the right ankle joint (ipsilateral joint) once a week for 4 weeks. The joint swelling in 0, 4, 8, 12 weeks was photographed (**A**). Scale bar, 5 mm. (**B**) Severity of arthritis presented as arthritis scores. Arrows are CFA treatments. ***p < 0.001, compared with 0 week by one-way ANOVA. (**C**) Diameters of ipsilateral (ipsi) and contralateral (contra) ankle joints. ***p < 0.001, ipsilateral vs contralateral by two-way ANOVA. (**D**) Threshold of paw withdrawal (PWT) to mechanical stimuli measured at weekly intervals in CFA-injected or uninjected mice (control). Data are mean ± SEM of n = 6 mice with CFA injection and n = 3~8 mice without injection. ***p < 0.001, ipsilateral vs contralateral; ^###^p < 0.001, CFA-injected vs uninjected by two-way ANOVA. (**E**) Serum level of TNFα. ^#^p < 0.05, compared with 0 weeks by nonparametric Mann-Whitney U test. (**F**) Serum level of IL-6. *p < 0.05, compared with 0 weeks by nonparametric Mann-Whitney U test. (**G**) pH value in synovial fluid in arthritic mice. Data are mean ± SEM. *p < 0.05, **p < 0.01, compared with 0 weeks by one-way ANOVA. qRT-PCR analysis of gene expression in ipsilateral DRG (**H**) and in contralateral DRG (**I**). Data are mean ± SEM. *p < 0.05, **p < 0.01, ***p < 0.001, compared with 0 weeks by one-way ANOVA.

The arthritic mouse model was established in ICR mice. Injected joints swelled after CFA injection as compared with non-injected joints and kept swelling for 12 weeks (Fig. 1A). In the first week post-injection, the mean arthritis score (measuring joint, toe, and paw swelling of 4 limbs) was 15.0 ± 3.0 (p = 0.000423, compared to 0 weeks, maximum scores 60); the severity of joint swelling increased over time and peaked at 4 weeks (mean 30.8 ± 5.6, p < 0.00001, compared to 0 weeks), remained stable for 8 weeks, and slightly declined at 12 weeks (26.0 ± 6.4, p < 0.00001, compared to 0 weeks) (Fig. 1B). After the first injection, the mean diameter of the injected joint increased and peaked at 4 weeks (5.68 ± 0.16 mm at 4 weeks vs 3.29 ± 0.08 mm at 0 weeks, p < 0.00001) (Fig. 1C). The mean joint diameter slightly declined after 4 weeks but remained swollen until 12 weeks (4.52 ± 0.13 mm, p < 0.00001). Joint diameter did not change much on the contralateral side, although contralateral toes and paws were swollen. In the first week, bilateral hyperalgesia for mechanical stimuli developed (mean ipsilateral PWT = 0.13 ± 0.02 g, p < 0.00001 compared with contralateral 0.53 ± 0.04 g) and remained stable for 12 weeks (ipsilateral PWT = 0.12 ± 0.02 g, p < 0.00001, compared with contralateral PWT = 0.32 ± 0.05 g; p < 0.00001, compared with ipsilateral PWT of naïve mice) (Fig. 1D).

Serum levels of TNF-α and IL-6 were measured in arthritic mice at 0, 4, 8, 12 weeks. TNF-α level was increased and peaked at week 4 (p = 0.0376, Fig. 1E); IL-6 level gradually increased from week 4 (p = 0.0373 for 4 weeks, p = 0.0118 for 8 weeks and p = 0.0253 for 12 weeks, compared to 0 weeks, Fig. 1F). The data was consistent with the previous suggestion that continuous IL-6 production is significant at the onset of chronic inflammatory disease^16^. High [H^+^] concentration in synovial fluid is associated with high RA disease activity^3^. Arthritic mice showed high mean [H^+^] concentration in synovial fluid at 4 weeks [662.6 ± 183.97 nmol/L, pH 6.18 ± 0.14 (p = 0.0138 compared to 0 weeks: ipsilateral 84.63 ± 21.53 nmol/L, pH 7.07 ± 0.13; contralateral 95.22 ± 17.6 nmol/L, pH 7.02 ± 0.09)] and 8 weeks [pH 6.17 ± 0.25 (p = 0.0069 compared to 0 weeks)] (Fig. 1G). At 12 weeks, mean [H^+^] concentration slightly reversed, to 290.07 ± 155.17 nmol/L (pH 6.53 ± 0.34). Besides the injected joint, the contralateral joint also showed slightly increased [H^+^] concentration. Acidosis in joints likely induces bilateral hyperalgesia.

Because a local acidosis signal is mediated by proton-sensing receptors, we examined gene expression in arthritic mice and found upregulated expression of the proton-sensing genes ASIC3 and TDAG8 in ipsilateral DRG. ASIC3 level was increased at 4 (p = 0.0131) and 12 weeks (p = 0.01602, compared to 0 weeks) and TDAG8 level at 12 weeks (p < 0.00001, compared to 0 weeks) (Fig. 1H). In contralateral DRG, expression levels of ASIC3 and TRPV1 genes were decreased at 12 weeks but TDAG8 remained unchanged (Fig. 1I).

**Figure 2:**
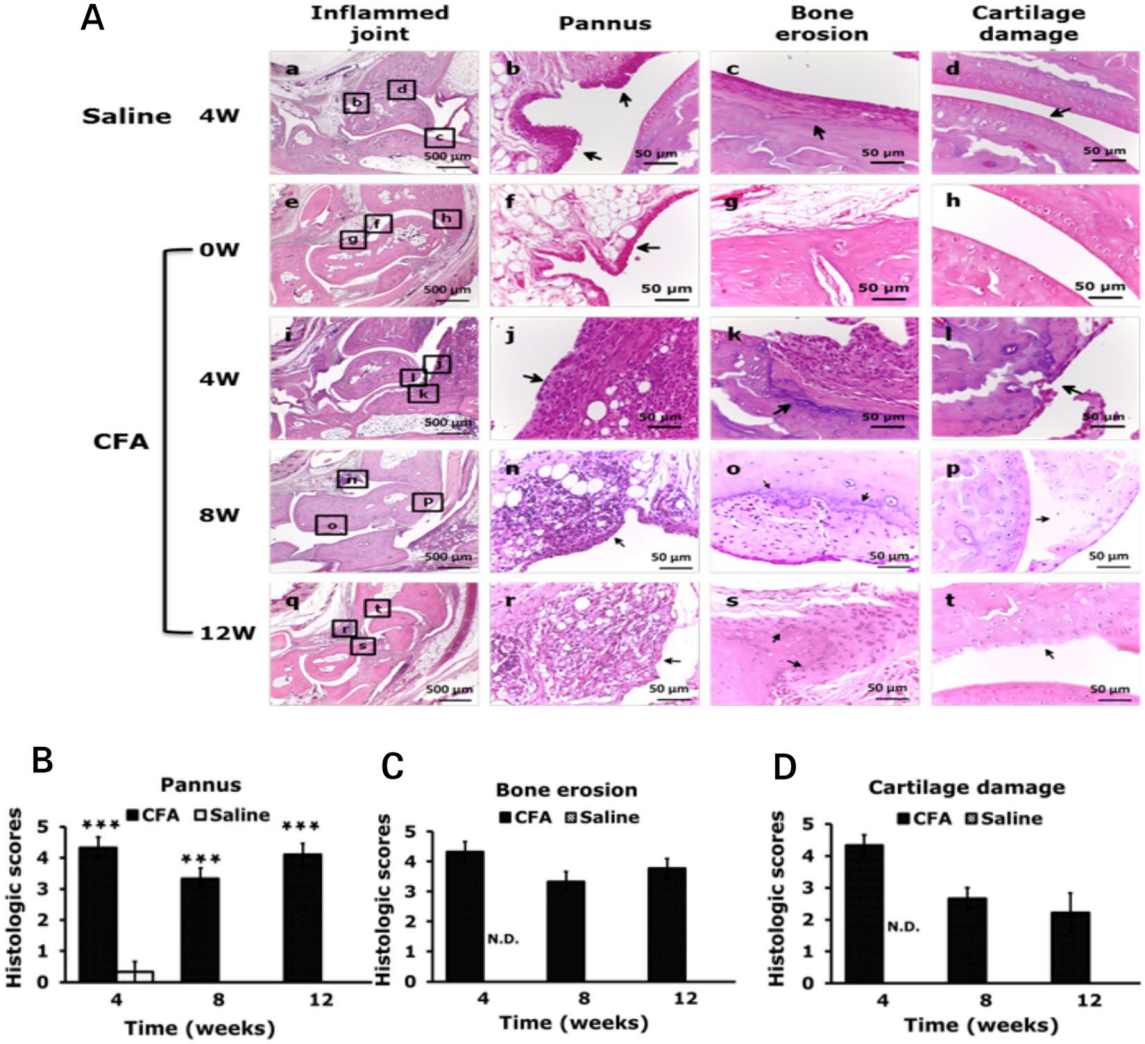
Histology of ipsilateral side joints in arthritic ICR mice. Samples of joints from arthritic mice at 0, 4, 8, 12 weeks stained with hematoxylin and eosin are shown in (**A**). Data are mean ± SEM severity score for pannus (**B**), bone erosion (**C**), and cartilage damage (**D**). ***p < 0.001, compared with 4-week saline-injected mice by nonparametric Mann-Whitney U test. Scores for bone erosion and cartilage damage in 4-week saline-injected mice were non-detectable (N.D.).

Histopathological examination of ankle joints of control mice (4-week saline injection) or before CFA injection (0 weeks) revealed a clear space between the bones and synovial membrane, with no inflammation and no bone erosion and cartilage damage (Fig. 2a–h). After CFA injection, synovial lining cells proliferated (pannus, p = 0.0005 for 4 weeks, p < 0.0000001 for 8 and 12 weeks, compared with saline-treated 4 weeks) and immune cells infiltrated into synovial fluid; tarsal bones (distal tibia, talus, navicular or calcaneous) were eroded; and cartilage was damaged (Fig. 2i–t). Although the arthritis scores were slightly decreased at 12 weeks, synovial inflammation was still severe at 12 weeks (Fig. 2q,r,B), with severe infiltration of immune cells into synovial fluid. Tarsal bones were increasingly eroded for 12 weeks (Fig. 2C), but cartilage damage was reduced at 12 weeks (Fig. 2D).

These results suggested that repeated CFA unilateral injection caused local acidosis, increased expression of proton-sensing genes, long-lasting inflammation and long-term mechanical hyperalgesia in bilateral sites.

### ASIC3-deficient mice show reduced arthritis-induced hyperalgesia in the late phase

**Figure 3:**
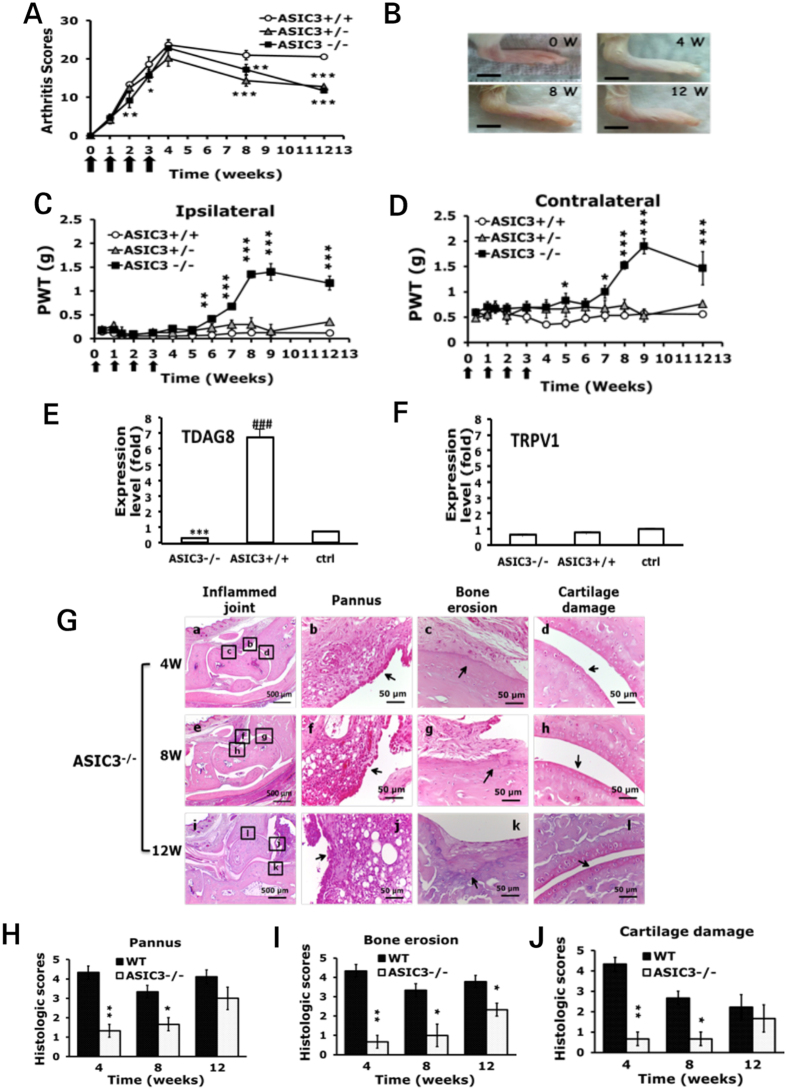
Arthritic ASIC3^−/−^ mice show reduced mechanical hyperalgesia in the late phase. (**A**) Arthritis scores in ASIC3^+/+^, ASIC3^+/−^ and ASIC3^−/−^ mice. *P < 0.05, **P < 0.01, ***p < 0.001, compared with ASIC3^+/+^ mice by two-way ANOVA. (**B**) Joint swelling in 0, 4, 8, 12 weeks in ASIC3^−/−^ mice. (**C**,**D**) PWT measured at weekly intervals after the first CFA injection. Data are mean ± SEM of total tested mice (n = 6 for ASIC3^+/+^, n = 8 for ASIC3^+/−^, n = 8 for ASIC3^−/−^ group). *p < 0.05, **P < 0.01, ***p < 0.001, ASIC3^−/−^ vs ASIC3^+/+^ by two-way ANOVA. (**E**,**F**) qRT-PCR analysis of TDAG8 or TRPV1 gene expression in ipsilateral DRG at 12 weeks of ASIC3^+/+^ or ASIC3^−/−^ mice, or at 0 weeks of ASIC3^+/+^ mice (ctrl). ***p < 0.001, ASIC3^-/-^ vs ASIC3^+/+^; ^###^p < 0.001, ASIC3^+/+^ vs ctrl by one-way ANOVA. (**G**) Samples of joints at 4, 8, 12 weeks stained with hematoxylin and eosin are shown in (a-l). Data are mean ± SEM severity score for synovial inflammation (**H**), bone erosion (**I**), and cartilage damage (**J**). *p < 0.05, **P < 0.01, comparing ASIC3^−/−^ vs wild type (WT) by nonparametric Mann-Whitney U test.

Because ASIC3 gene expression was upregulated after intra-articular CFA injection, we investigated whether loss of ASIC3 gene reduced CFA-induced arthritis. ASIC3^+/+^ mice, like wild-type mice, showed long-term inflammation with CFA injection, with a peak at 4 weeks (mean 39.4 ± 1.8% and 37.1 ± 3.1%) and long-lasting hyperalgesia for 12 weeks (Fig. 3A,B). ASIC3^+/−^ mice showed a similar pattern as ASIC3^+/+^ mice in arthritis scores and hyperalgesia, except that arthritis scores in ASIC3^+/−^ mice decreased at 8 weeks (Fig. 3A). ASIC3^−/−^ mice showed less severe arthritis at 2 weeks (p = 0.043, compared to ASIC3^+/+^) but a similar peak level as ASIC3^+/+^ mice at 4 weeks. However, arthritis severity decreased at 8 and 12 weeks for deficient mice (p = 0.0099 for 8 weeks; p < 0.00001 for 12 weeks) (Fig. 3A). Swelling of paws and joints was less in ASIC3^−/−^ than ASIC3^+/+^ mice (Fig. 3B). Mechanical hyperalgesia was bilateral in ASIC3^−/−^ mice after the first injection but was gradually reduced from 6 weeks on ipsilateral side (p = 0.0036, Fig. 3C) and from 7 weeks on contralateral side (p = 0.0187, Fig. 3D). Depletion of ASIC3 may have reduced arthritis-induced chronic pain in the late phase (after 6 weeks). RA mice had an increased expression of TDAG8 gene at 12w (Fig. 1H). Depletion of ASIC3 inhibited the increase of TDAG8 gene expression (p = 0.00002, compared to ASIC3^+/+^) (Fig. 3E), but did not change TRPV1 gene expression (Fig. 3F).

Consistent with a decrease in arthritis scores, the severity of synovial inflammation, bone destruction, and cartilage damage was attenuated in ASIC3^−/−^ mice from 4 weeks (Fig. 3G–J), except that synovial inflammation and cartilage damage were not significantly reduced at 12 weeks (Fig. 3H,J).

### TRPV1-deficient mice show reduced arthritis-induced hyperalgesia in the late phase

**Figure 4:**
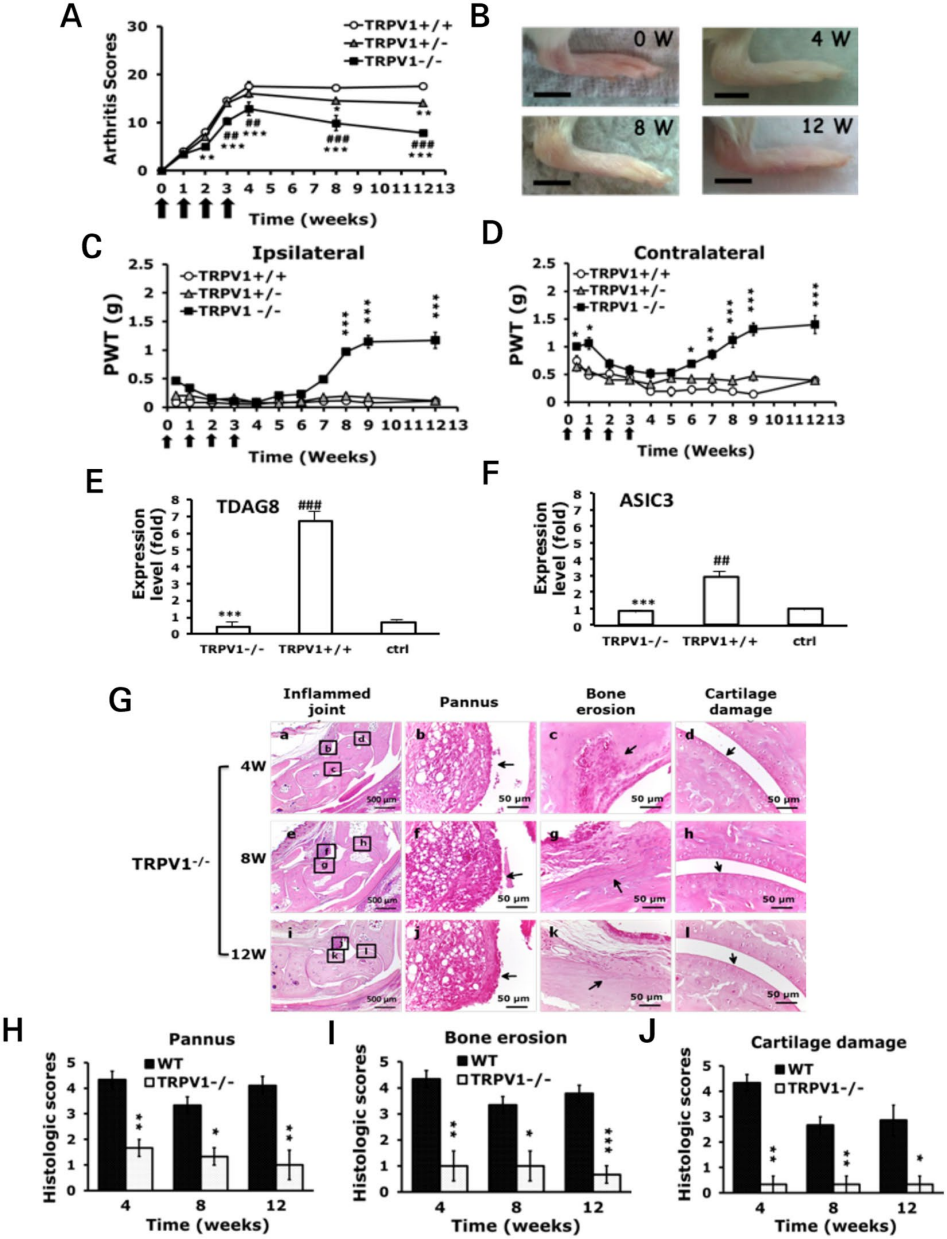
Arthritic TRPV1^−/−^ mice show reduced mechanical hyperalgesia in the late phase. (**A**) Arthritis scores in TRPV1^+/+^, TRPV1^+/−^ and TRPV1^−/−^ mice. *p < 0.05, ^##^**P < 0.01, ^###^***p < 0.001: * TRPV1^−/−^ or TRPV1^+/−^ vs TRPV1^+/+^, ^#^TRPV1^+/−^ vs TRPV1^−/−^ by two-way ANOVA. (**B**) Joint swelling at 0, 4, 8, 12 weeks in TRPV1^−/−^ mice. (**C**,**D**) PWT measured at weekly intervals after the first CFA injection. Data are mean ± SEM of total tested mice (n = 7 for TRPV1^+/+^, n = 6 for TRPV1^+/−^ and n = 7 for TRPV1^−/−^ group). *p < 0.05, **P < 0.01, ***p < 0.001: TRPV1^−/−^ vs TRPV1^+/+^ by two-way ANOVA. (**E**,**F**) qRT-PCR analysis of TDAG8 or ASIC3 gene expression in ipsilateral DRG at 12 weeks of TRPV1^+/+^ or TRPV1^−/−^ mice, or at 0 weeks of TRPV1^+/+^ mice (ctrl). ***p < 0.001, TRPV1^-/-^ vs TRPV1^+/+^; ^###^p < 0.001, TRPV1^+/+^ vs ctrl by one-way ANOVA. (**G**) Samples of ipsilateral joints at 4, 8, 12 weeks stained with hematoxylin and eosin are shown in (a-l). Data are mean ± SEM severity score for synovial inflammation (**H**), bone erosion (**I**), and cartilage damage (**J**). *p < 0.05, **P < 0.01, comparing TRPV1^−/−^ vs WT by nonparametric Mann-Whitney U test.

**Figure 6:**
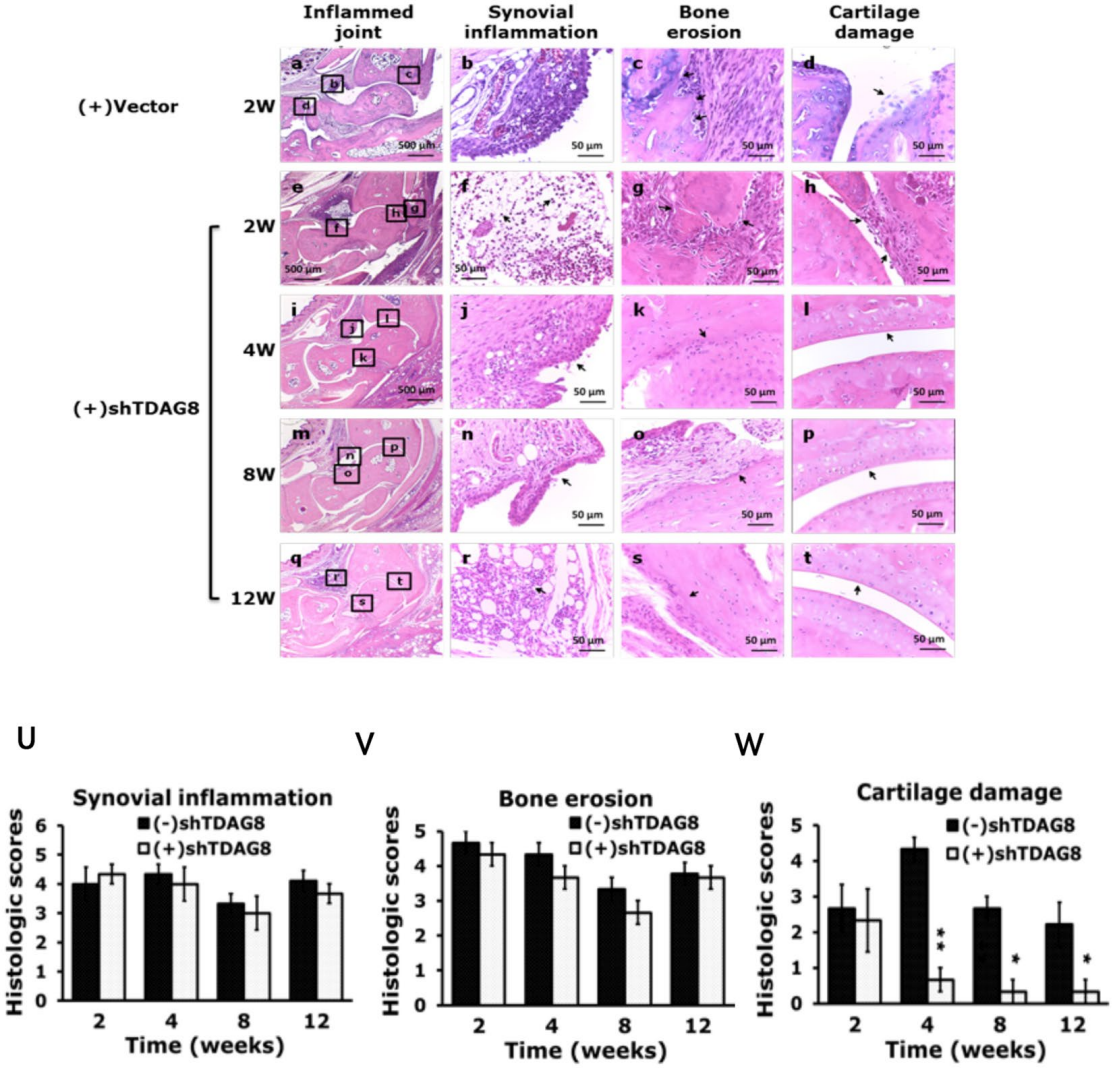
Histology of ipsilateral joints in Arthritic TDAG8 knockdown mice. Samples of joints from shTDAG knockdown mice at 0, 4, 8, 12 weeks stained with hematoxylin and eosin are shown in (a-t). Data are mean ± SEM severity score for pannus (U), bone erosion (V), and cartilage damage (W). *p < 0.05, **P < 0.01, comparing (+)shTDAG8 vs (−)shTDAG8 by nonparametric Mann-Whitney U test.

Similar experiments of TRPV1-deficient mice explored the role of TRPV1 in arthritis. TRPV1^+/+^ and TRPV1^+/−^ mice, like wild-type ICR mice, showed long-term inflammation: the mean arthritis scores increased with time and peaked at 4 weeks (Fig. 4A). Mean arthritis scores increased in TRPV1^−/−^ mice over time, but the severity of arthritis was largely reduced from week 2 as compared with TRPV1^+/+^ mice (p = 0.007 at 2 weeks, p < 0.000001 at 12 weeks) (Fig. 4A). TRPV1^−/−^ mice showed less severe swelling of paws and joints (Fig. 4B). Mechanical hyperalgesia in TRPV1^+/+^ and TRPV1^+/−^ mice, like wild-type mice, was bilateral after the first injection and lasted for 12 weeks, whereas bilateral mechanical hyperalgesia in TRPV1^−/−^ mice started to decrease from 8 weeks on the ipsilateral side (p < 0.000001, compared with TRPV1^+/+^, Fig. 4C) and 6 weeks on the contralateral side (p = 0.0416, Fig. 4D). Depletion of TRPV1 may have reduced arthritis-induced chronic pain in the late phase (after 8 weeks). In RA mice, expression of TDAG8 gene was increased at 12 weeks and expression of ASIC3 gene was increased at 4 and 12 weeks (Fig. 1H). Depletion of TRPV1 inhibited the increase in gene expression of ASIC3 (p = 0.00096, compared to TRPV1^+/+^) and TDAG8 (p = 0.00002, compared to TRPV1^+/+^) at 12 weeks (Fig. 4E,F). Consistent with a decrease in arthritis scores, pannus, bone destruction, and cartilage damage were significantly reduced in TRPV1^-/-^ mice at 4, 8, 12 weeks (Fig. 4G–J); was not reduced in TRPV1^+/-^ mice at 4 weeks (suppl Fig. 1).

### TDAG8-knockdown mice show reduced arthritis-induced hyperalgesia in the early phase, whereas TDAG8 knockout mice show attenuated hyperalgesia in both acute and chronic phases

**Figure 5:**
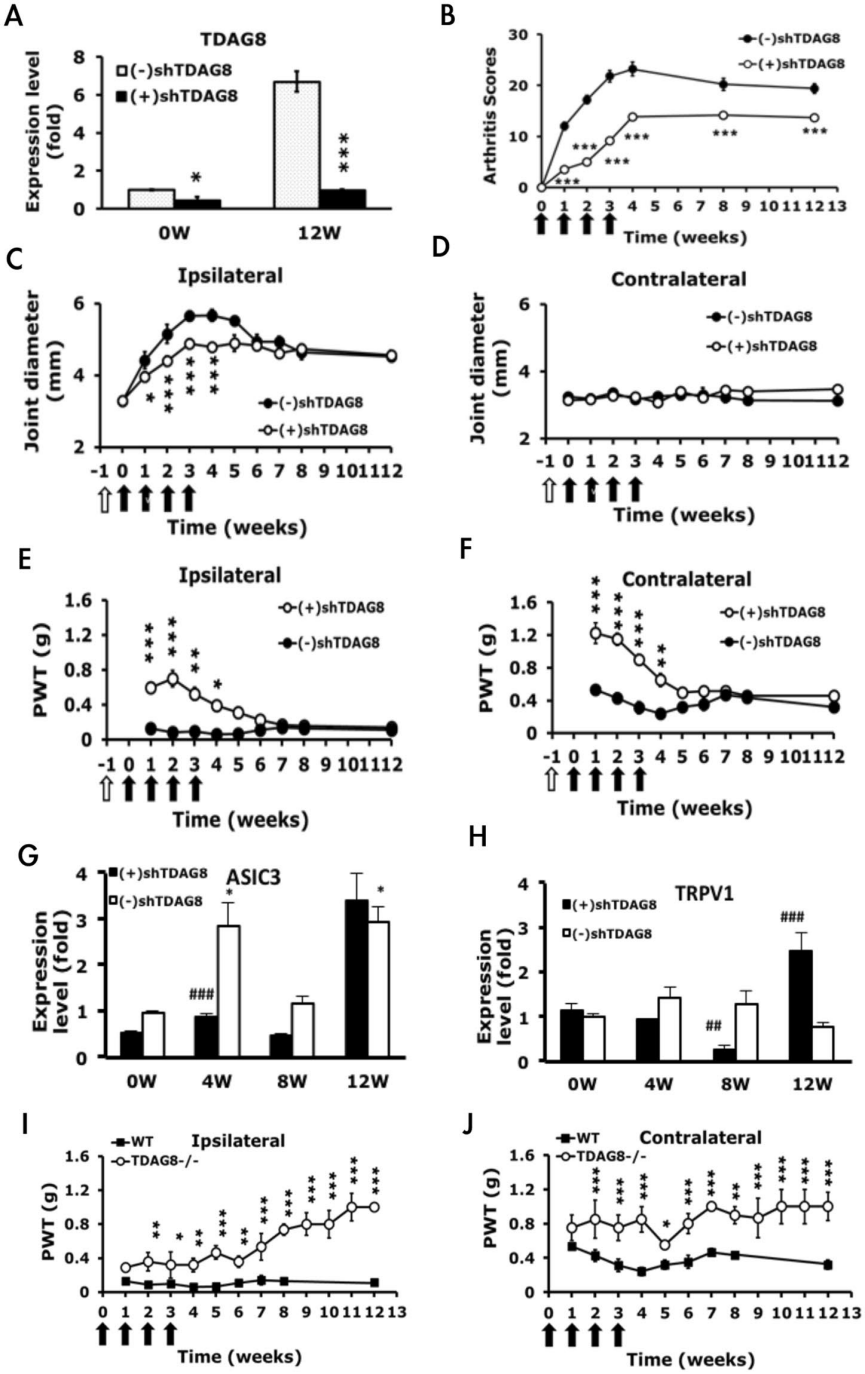
Arthritic TDAG8 knockdown mice show reduced mechanical hyperalgesia in the early phase, but TDAG8 knockout mice attenuated hyperalgesia in both early and late phases. (**A**) TDAG8 gene expression before (0 weeks) or after CFA injection (12 weeks) in L1-5 DRG. *P < 0.05, ***p < 0.001: shTDAG8-injected [(+)shTDAG8] vs vector-injected mice [(−)shTDAG8] by one-way ANOVA. (**B**–**D**) Arthritis scores (**B**) and joint diameter (**C**,**D**) in TDAG8-knockdown mice. *p < 0.05, ***p < 0.001: (+)shTDAG8 vs (−)shTDAG8 by two-way ANOVA. (**E**,**F**) PWT measured at weekly intervals after the first CFA injection. Data are mean ± SEM of total tested mice (n = 6 for (−)shTDAG8, and n = 8 for (+)shTDAG8). *p < 0.05, **P < 0.01, ***p < 0.001: (+)shTDAG8 vs (−)shTDAG8 by two-way ANOVA. (**G**,**H**) qRT-PCR analysis of ASIC3 or TRPV1 gene expression in ipsilateral L1-5 DRG of TDAG8 knockdown mice. Data are mean ± SEM. *p < 0.05, compared with 0 weeks; ^##^p < 0.01, ^###^p < 0.001, compared with WT control by one-way ANOVA. (**I**,**J**) PWT measured at weekly intervals after the first CFA injection in TDAG8^−/−^ or WT mice. Data are mean ± SEM of total tested mice (n = 4 for TDAG8^−/−^, n = 6 for WT) *p < 0.05, **P < 0.01, ***p < 0.001: TDAG8^−/−^ vs WT by two-way ANOVA.

TDAG8 gene expression was upregulated at 12 weeks (Fig. 1E), so we examined whether suppression of TDAG8 expression could reduce arthritis-induced hyperalgesia. We used an shRNA specific to TDAG8 (shTDAG8) to suppress TDAG8 gene expression^17^. Mice were intraplantarly pre-injected with shTDAG8 before arthritis was induced 7 days later. Joint afferents were traced by intra-articular injection of fluorogold (FG). shTDAG8 was expressed in FG-positive neurons (suppl Fig. 2). shTDAG8 reduced TDAG8 expression by 50% in DRG before CFA injection (0 weeks) and continued to inhibit expression up to 12 weeks (Fig. 5A). TDAG8 protein level was also reduced (suppl Fig. 2), as previously described^17^. Arthritis scores were reduced from the first week and lasted for 12 weeks (p < 0.000001, compared to (−)shTDAG8) (Fig. 5B). The joint diameter on the ipsilateral side was significantly decreased in the first 4 weeks (Fig. 5C,D). Arthritis-induced hyperalgesia was markedly attenuated from the first week in both ipsilateral (p < 0.0009) and contralateral (p < 0.000001) sides of TDAG8-knockdown mice (Fig. 5E,F) and continued for 4 weeks. In previous study, TDAG8 knockout or knockdown mice exhibited shortened inflammation or acid-induced hyperalgesia^17^. However, TDAG8 knockdown did not shorten arthritis-induced hyperalgesia in our RA model. Suppression of gene expression (50%) in this case was likely not sufficient to attenuate chronic hyperalgesia of RA pain. In TDAG8 knockdown mice, ASIC3 and TRPV1 gene expression was maintained at a low level from the beginning to 8 weeks but not at 12 weeks (Fig. 5G,H). It could partly explain that TDAG8 knockdown did not attenuated chronic hyperalgesia because ASIC3 and TRPV1 expression was higher at week 12. To address whether TDAG8 knockdown (50% reduction in gene expression) is insufficient to affect chronic hyperalgesia, similar experiments were performed in TDAG8^−/−^ mice. TDAG8 deficiency attenuated hyperalgesia in the acute phase and also attenuated chronic hyperalgesia of RA pain (Fig. 5I,J).

Although TDAG8 knockdown reduced arthritis scores and joint swelling, TDAG8 knockdown did not significantly prevent synovial inflammation and bone destruction but did prevent cartilage damage (Fig. 6A–W).

### ASIC3- or TRPV1-deficiency or shTDAG8 knockdown reduces synovial CD68+ macrophages at 12 weeks

**Figure 7:**
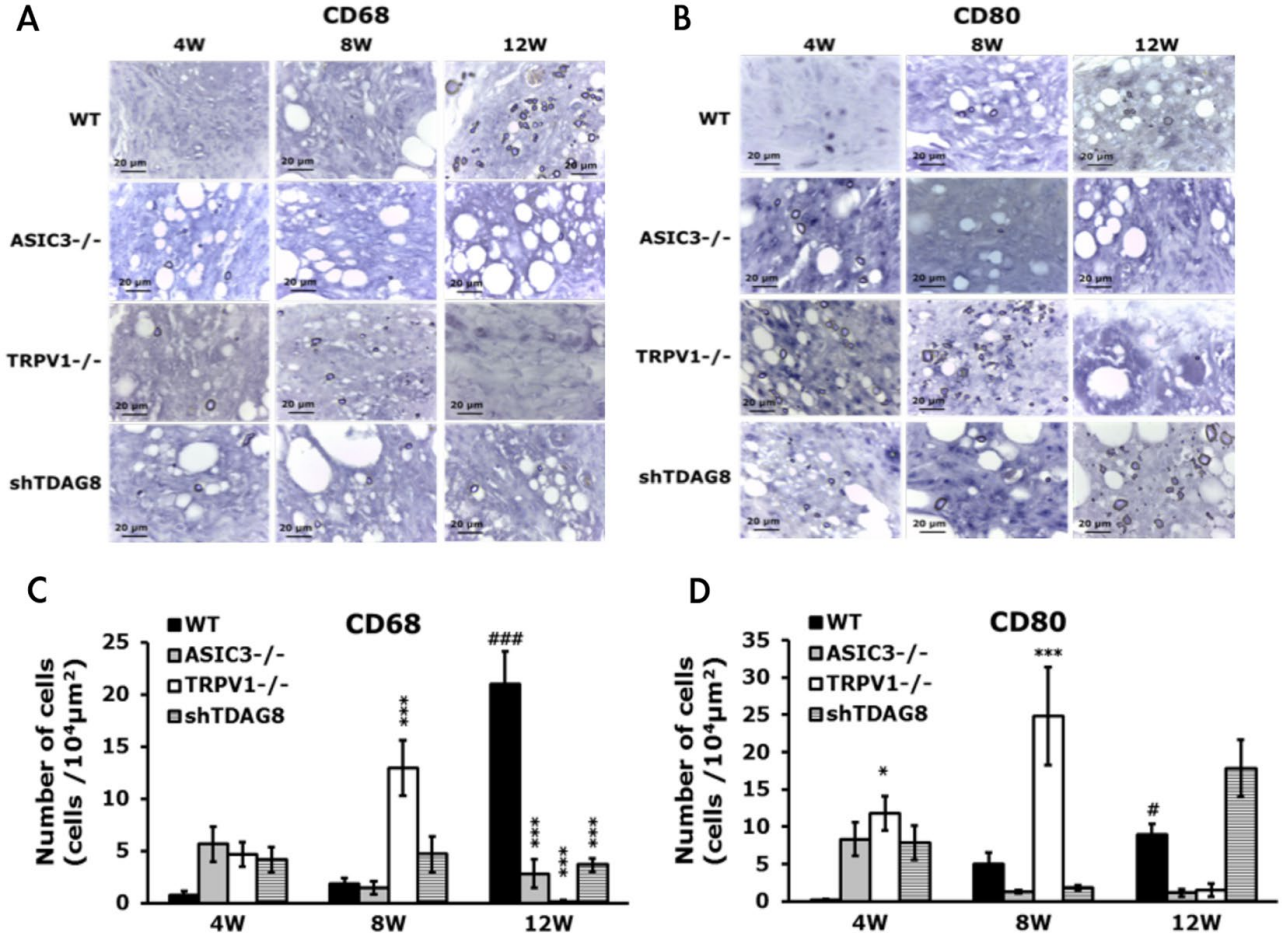
ASIC3- or TRPV1-deficiency or shTDAG8 knockdown reduces synovial CD68^+^ macrophages at 12 weeks Samples of ipsilateral joints from WT, ASIC3^−/−^, TRPV1^−/−^, shTDAG knockdown mice at 4, 8, 12 weeks were stained with CD68 (**A**,**C**) or CD80 (**B**,**D**) antibodies. Synovial sublining regions are shown in (**A**,**B**). Magnification, 100x. The number of cells in 100 × 100 μm^2^ was calculated and presented as histograms (**C**,**D**). ^#^p < 0.05, ^###^p < 0.001, compared with 4w; *p < 0.05, ***p < 0.001, compared with WT by one-way ANOVA.

Macrophages play a key role in the pathogenesis of RA. Synovial macrophage number can be used as a biomarker for disease activity. TDAG8, ASIC3, and TRPV1 influence arthritis scores and RA-induced hyperalgesia. Whether these genes affect synovial macrophages remains unclear. CD68, a marker of synovial macrophages, and CD80, a marker of M1 macrophages, were used to address this issue. CD68^+^ macrophages were only found in synovial sublining region, but CD80^+^ macrophages were found in synovial intimal lining, sublining, and cartilage (Fig. 7A,B). In RA mice, the number of CD68^+^ and CD80^+^ macrophages was significant increased at 12 weeks (Fig. 7C,D). TDAG8 knockdown reduced the number of CD68^+^ macrophages at 12 weeks, but not altered the number of CD80^+^ macrophages (Fig. 7C,D). ASIC3 deletion reduced the number of CD68^+^ macrophages at 12 weeks and slightly reduced the number of CD80^+^ macrophages (Fig. 7C,D). TDAG8 immunoreactivity was found in synovial sublining, while ASIC3 immunoreactivity was found in synovial sublining and cartilage (suppl Fig. 3). It could partially explain why ASIC3 deficiency affects CD68^+^ and CD80^+^ macrophages, but TDAG8 knockdown only influence CD68^+^ macrophages. In contrast to ASIC3 and TDAG8, TRPV1 deletion increased the number of CD68^+^ macrophages at 8 weeks, but reduced the number at 12 weeks (Fig. 7C). Similar results were found in CD80^+^ macrophages (Fig. 7D).

## Discussion

We provide new insights into the roles of proton-sensing receptors in the RA pain mechanism and disease progression. We established the arthritis model in ICR mouse by CFA injection into the tibiotarsal joint once a week for 4 weeks. Arthritic mice showed long-term joint inflammation and long-lasting bilateral hyperalgesia for at least 12 weeks. Similar to clinical RA patients, repeated CFA injection in mice induced high [H^+^] concentration in synovial fluid in the 4 weeks, a continued serum IL-6 production, and an increased number of synovial macrophages. Two proton-sensing genes, ASIC3 and TDAG8 were upregulated after CFA injection. Deletion of ASIC3 or TRPV1 reduced disease progression from the early phase (starting at week 2) but did not attenuate bilateral hyperalgesia until weeks 6 or 8. In contrast, deletion of TDAG8, a proton-sensing GPCR, not only suppressed bilateral hyperalgesia in the acute phase but also attenuated chronic phase. Continuous suppressed expression of all three genes could be essential to attenuated chronic phase of RA pain.

We successfully established the arthritis model in the ICR mouse. Similar to results in C57BL/6 mice^18^, ICR mice with CFA injection showed long-term joint inflammation with severe bone erosion and cartilage damage, accompanied by long-lasting bilateral hyperalgesia after repeated injection. The inflammation and hyperalgesia lasted for at least 12 weeks. Redness and swelling were found in 4 limbs (paw, digits, joint) and more severe in the injected tibiotarsal joint. Joint diameter was significantly increased in injected joints but not the contralateral joint. However, the contralateral joint was swollen, despite no apparent increase in joint diameter. The arthritis scores peaked at 4 weeks (50% of the maximum) and lasted for 12 weeks. Given that the maximum arthritis score for each limb was 25%, 50% of arthritis scores indicated that inflammation occurred in 4 limbs, not just one limb. Joint pathology showed severe synovial inflammation, bone erosion and cartilage damage from week 4 that continued for 12 weeks in the injected joint but not the contralateral joint. However, a continuous serum IL-6 production and an increased number of synovial macrophages (CD68^+^ macrophages) marked the disease in the chronic inflammatory state. We found high [H^+^] concentration in synovial fluid from both ipsilateral and contralateral tibiotarsal joints at week 4 after CFA injection. In the clinical study by Farr *et al*.^3^, only RA patients but not osteoarthritis (OA) or other arthritis patients showed high [H^+^] concentration in synovial fluid. Our arthritic mice developed chronic and systemic inflammation that may explain why contralateral paw also developed hyperalgesia because contralateral hyperalgesia (mirror-image pain) can be triggered by a high level of unilateral inflammation (high levels of TNFα and cytokines) through central or peripheral glial cell activation^19, 20^. Therefore, our arthritis model could reproduce some of possible mechanisms at play in RA, rather than OA or other arthritis. It is an animal model of chronic joint inflammation and pain, as compared with the collagen/LPS model, and suitable for therapeutic application for RA pain.

Consistent with previous studies of TRPV1 in C57BL/6 mice^11–13^, our deletion of TRPV1 gene attenuated synovial inflammation, bone erosion, and cartilage damage in the joint in the early disease phase, which was maintained at the later phase. Because TRPV1 is expressed in fibroblast-like synoviocytes, TRPV1 expression is increased after inflammation^21–23^, and TRPV1 deletion inhibited synovial macrophages (M1 type), TRPV1 deletion may block the pro-inflammatory function, thereby reducing synovial inflammation. Protection of bone and cartilage destruction could be due to inhibiting synoviocyte invasion or synoviocyte-released signals of receptor activator of nuclear factor-kB ligand (RANKL)^1^. Fibroblast-like synoviocytes also release inflammatory mediators to upregulate pain-related receptors (such as NK1) in joint afferents^21^, which leads to pain and hyperalgesia. TRPV1 regulates synovial inflammation from the beginning of disease, but TRPV1 deficiency affected RA-associated pain beginning at the later phase (>8 weeks). TRPV1 may not regulate the initiation of arthritic pain but rather regulates the establishment of chronic phase of arthritic pain. A previous study of muscle pain proposed that ASIC3 and TRPV1 mediate acidosis signals to establish hyperalgesic priming in the muscle pain model^24^. In agreement, we found that TRPV1 had a priming effect on RA-associated hyperalgesia. This priming effect could be attributed to prevention of an increase in expression of ASIC3 and TDAG8 genes. The role of TRPV1 in chronic hyperalgesia induced by arthritis was not reported previously because previous studies examined only the acute phase (<5 weeks) and used different models^10, 11, 13^. Our model represented a model of chronic joint inflammation and pain, with possible mechanisms at play in RA, which could explain the different observations found between our model and other models.

ASIC3 is expressed in joint afferents^8^, macrophages^25^, synovial cells and chondrocytes^9^. ASIC3 regulates synovial inflammation, bone erosion, cartilage damage, and pain, but its effects are more complex than TRPV1. Here, similar to TRPV1, ASIC3 deficiency prevented synovial inflammation, bone erosion and cartilage from the disease beginning (4 weeks), but ASIC3 deficiency affected RA-associated pain starting from the later phase (>6 weeks). Thus, ASIC3 is also involved in establishing hyperalgesic priming, as was suggested previously in a muscle model^22^. This observation was not reported before because most studies focused on the acute phase (<4 weeks) in different arthritis models^6, 7, 10^.

Whether ASIC3 deficiency increases inflammation is arguable from previous studies using different arthritis models. In the collagen/LPS model, ASIC3 deficiency increased inflammation^7^ but attenuated inflammation in the OA model^10^. In our RA model, ASIC3 deletion reduced pannus from weeks 4 to 8. At 12 weeks, ASIC3 deficiency seemed to not fully suppress pannus, although it reduced arthritis scores and synovial macrophages (M1 type). ASIC3 expressed in synoviocytes seems to mediate inflammatory cytokines or other substances to increase nerve innervation to synovium, thereby leading to pain and inflammation^9^. ASIC3 also mediates acid-induced endocytosis and maturation of macrophages to promote innate inflammation^25^. Macrophages have proinflammatory and anti-inflammatory phenotypes to mediate synovial inflammation and joint destruction^26^. Although RA is considered to exhibit more inflammatory responses than OA, it still features anti-inflammatory responses, with macrophages playing a large part. Thus, deletion of ASIC3 reduced synovial inflammation from the disease beginning because of the inhibition of the pro-inflammatory macrophage function but with less reduction in the later phase of inflammation (inhibition of anti-inflammatory macrophage function).

Extracellular high [H^+^] concentration inhibits matrix synthesis by chondrocytes, affects biosynthetic ability of chondrocytes, and even induces chondrocyte apoptosis^27–29^. Inhibition of ASICs by amiloride prevents injury of chondrocytes induced by acid^27, 30, 31^. ASIC3 acts as a pH sensor in chondrocytes to regulate hyaluronan expression^9^. Inhibition of ASIC3 by APETx2 protected against cartilage destruction in an OA model^10^. These results support our observation that ASIC3 deficiency protected cartilage damage.

In contrast to ASIC3 and TRPV1, TDAG8 contributed to pain in the acute and chronic phase. TDAG8 was found involved in inflammatory pain and bone cancer pain^17, 32, 33^. As well, TDAG8 participates in initiating hyperalgesia and establishing hyperalgesic priming in a dual-acid or CFA-induced inflammatory model^17, 34^. However, here we found that TDAG8 knockdown reduced hyperalgesia only in the acute phase (<4 weeks) but did not observe the priming effect of TDAG8. The efficiency of shTDAG8 knockdown (50% reduction) may not be sufficient to observe the priming effect in an RA model. Using TDAG8 knockout mice, we demonstrated that TDAG8 deficiency indeed attenuated chronic hyperalgesia in our RA model, so TDAG8 may be involved in hyperalgesic priming in this RA model. RA model is a systemic and chronic inflammatory pain model but CFA is a subacute inflammatory pain model. Thus, 50% reduction in TDAG8 gene expression could be insufficient to attenuate chronic hyperalgesia resulted from serve inflammation (such as RA model). A failure to attenuate hyperalgesia in the late phase is not due to retrograde transport of shTDAG8 plasmids by intraplantar injection. By intra-articular injection of fluorogold, we found the majority of shTDAG8 plasmids were expressed in fluorogold-labeled neurons.

In ASIC3 and TRPV1 knockout mice with attenuated chronic hyperalgesia, expression of TDAG8, ASIC3, and TRPV1 genes were eliminated or at low levels from the beginning until 12 weeks. Given that reduced hyperalgesia starting from 6 weeks in ASIC3 knockout mice but starting from 8 weeks in TRPV1 knockout mice, it seems that the starting time for ASIC3 to regulate hyperalgesia is earlier than that for TRPV1. TDAG8 knockdown greatly inhibited ASIC3 expression at 4 weeks but inhibited TRPV1 expression at 8 weeks. This finding seems to agree with ASIC3 regulation in hyperalgesia starting earlier than TRPV1 regulation. However, at 12 weeks, the inhibitory effect on ASIC3 and TRPV1 expression had disappeared, which may explain why TDAG8 knockdown mice did not display reduced hyperalgesia in the late phase.

TDAG8 gene expression in RA mice is not increased until 12 weeks, but the TDAG8 knockdown or knockout mice showed reduced hyperalgesia earlier. It is likely that a moderate level of TDAG8 is sufficient to initiate hyperalgesia. However, an increased TDAG8 level in the late phase could be important to maintain RA progression. In contrast, ASIC3 has two phases to increase gene expression. The first phase could be related to establishment of hyperalgesic priming at 4 weeks; while the second phase could be related to maintenance of chronic hyperalgesia at 12 weeks. In contrast to ASIC3 and TDAG8, a moderate level of TRPV1 could be sufficient to the establishment of hyperalgesic priming, so no increase in TRPV1 gene expression was found. Accordingly, TDAG8 plays a role in the initial phase of hyperalgesia and the initial regulation also affects the establishment of the chronic phase (priming effect). The establishment of chronic pain is attributed to TDAG8, ASIC3, and TRPV1 genes that regulating the late phase of hyperalgesia. Suppression of all three gene expression may lead to shortened hyperalgesia.

A previous study of TDAG8-deficient mice found that TDAG8 deficiency promoted arthritis^15^, but the authors did not assess arthritis-induced pain. Although TDAG knockdown reduced arthritis scores and joint swelling, the inhibitory effect seems not related to synovial inflammation because synovial inflammation was not inhibited with TDAG8 knockdown. Given that TDAG8 mediates acid-inhibited cytokine production in macrophages, T cells, neutrophils and microglia^35–37^ and that TDAG8 activation inhibits acidification-enhanced bone resorption^38^, TDAG8 activation should facilitate inflammation and bone erosion. Thus, synovial inflammation and bone erosion were not attenuated in TDAG8-knockdown mice. However, mice with TDAG8 knockdown mice showed a significant chondroprotection effect, even though synovial inflammation was not inhibited. Which mechanism TDAG8 mediates to protect cartilage damage is unclear. TDAG8 likely responds to acid signals to mediate acid-induced injury of chondrocytes. Suppression of TDAG8 prevents injury of chondrocytes.

## Conclusions

This study demonstrated that ASIC3, TRPV1 and TDAG8 are modulators of RA disease progression and RA-associated pain. Deletion of ASIC3 or TRPV1 prevented RA disease progression and establishment of hyperalgesic priming, thereby leading to attenuation of the chronic phase of RA pain (>6 weeks or >8 weeks). TDAG8 is involved in both the acute (<4 weeks) and chronic phase (>6 weeks) of RA pain. TDAG8 likely participates in initiation of hyperalgesia and TDAG8, ASIC3, and TRPV1 are essential to establish hyperalgesic priming. This is the first study to provide evidence that TDAG8, ASIC3 and TRPV1 regulate hyperalgesic priming of RA pain and that a transition from acute to chronic RA pain is at about 4 weeks. We bring new insights for the development of therapeutic treatments.

## Materials and Methods

### Animal arthritis model

Male and female ICR mice (8–12 weeks old) were purchased from BioLASCO Taiwan (Taipei) and housed 3–4 per cage under a 12-h light/dark cycle (lights on at 7:00) with food and water *ad libitum* in a temperature- and humidity-controlled environment at the National Central University. Care and use of mice conformed to the Guide for the Use of Laboratory Animals (US National Research Council), and the experimental procedures were approved by the local animal use committee (IACUC, National Central University, Taiwan). All behavioural testing was performed between 9:00 and 17:00. Efforts were made to minimize the number of animals used and their suffering. For gene expression, mice were placed in the euthanasia chamber and killed by introducing 100% CO_2_ with a fill rate of 20% to 30%/min. Mice were unconscious usually within 2 to 3 min. Dorsal root ganglia (DRG) were excised for RNA extraction.

ASIC3^−/−^, ASIC3^+/−^, and ASIC3^+/+^ mice (on an ICR background) were generated as described^39^. The genotyping primer sequences were for ASIC3^−/−^, 5′-attcaggctgcgcaactgtt/5′-tgtggtcccaggacttggta; and ASIC3^+/+^, 5′cacagctccaggaggagttgaa/5′-ccttgtgacgaggtaacaggta. TRPV1^−/−^ TRPV1^+/−^, and TRPV1^+/+^ mice on an ICR background were generated as described^40^. The genotyping primer sequences were for TRPV1^−/−^, 5′-cacgagactagtgagacgtg/5′-tcctcatgcacttcaggaaa; and TRPV1^+/+^, 5′-cctgctcaacatgctcattg/5′-tcctcatgcacttcaggaaa. TDAG8^−/−^ and TDAG8^+/+^ mice on a B6 background were generated as decribed^17^. The genotyping primer sequences were for TDAG8^−/−^, 5′-gaaccattagtttggctcatgtgactg/5′-cttgtgtcatgcacaaagtagatgtcc; and TDAG8^+/+^, 5′-cgaactctagctggcttttatccaataat/5′-gaaccattagtttggctcatgtgactg. TDAG8 knockdown mice were created by delivering a TDAG8-shRNA clone to DRG by intraplantar injection with microspheres of cationized gelatin (CG) (from Dr. Yasuhiko Tabata, Kyoto University, Japan)^41^. Mice were intraplantarly injected with 12.5 μg shTDAG8/CG (1:7.5) and at 7 d, injected with 5 μl CFA (5 μg) in the right ankle joint (ipsilateral joint) once a week for 4 weeks. Preparation of the shTDAG8/CG polyplex was as described^17, 42^. Briefly, plasmid DNA (shTDAG8) and CG were each heated to 65 °C for 10 min and separately diluted into 5% glucose. CG was added into plasmid DNA at a weight ratio of 7.5:1 and vortexed for 30 sec before injection in mice. The TDAG8-shRNA clone (TRCN0000027436) was from the Taiwan National RNAi Core Facility, Academia Sinica, Taiwan, and was subcloned into a pLKO.1-Cherry vector. The target sequence for TDAG8 was 5′-ccagccaacatcggatcttta.

Arthritis was induced in male or female mice by the method of Gauldie *et al*.^18^. Mice were injected with 5 μg CFA in the right ankle joint once a week for 4 weeks. Control mice were injected with saline. At different times post-injection, joint diameter was measured and arthritis and histologic scores and pain sensitivity were determined. In some experiments, mice were killed at various weeks and DRG were excised for measuring gene expression. Joint afferents was labelled by intra-articular injection of 5% fluorogold (5μL) into mice and at 7 d, CFA was injected into the joint. At 1week post CFA injection, DRG was isolated, frozen, and sectioned for immunostaining.

### Assessment of arthritis, histology and synovial pH values

After CFA injection, arthritis severity was assessed in the ankle/wrist and paw as grade 0 = normal, 1 = redness and mild swelling, 3 = moderate redness and swelling, 5 = severe redness and swelling. Toe swelling was scored as 0 = normal, 1 = swelling. Each limb was graded and given a maximum possible score of 15; the maximum score for each animal was 60.

At 0, 4, 8, and 12 weeks after CFA injection, tibiotarsal joints were excised, fixed in 10% formalin overnight, then transferred to 50% ethanol. Fixed tissues were decalcified, embedded in paraffin and sectioned with use of a microtome, then stained with hematoxylin and eosin (by the Taiwan Mouse Clinic, Taipei). Some sections were stained with CD80 (1:250, Biorbyt, UK), CD68 (1:100, Biorbyt) or anti-TDAG8 (1:200, custom by Genesis, Taiwan) antibodies, followed by alkaline phosphatase -conjugated goat anti-rabbit IgG (1:5000, Jackson Immunoresearch). Signals were developed by nitro-blue tetrazolium chloride and 5-bromo-4-chloro-3′-Indolyphosphate *p*-toluidine (Millipore). Some sections were stained with anti-ASIC3 (1:200, Alomone labs) or anti-TDAG8 antibodies, followed by FITC-conjugated anti-guinea pig IgG (1:250, Alomone labs) or DyLight 405-conjugated goat anti-rabbit IgG (1:300, Jackson Immunoresearch). Arthritic changes were scored on a scale of 0 to 5^43^. Synovial inflammation was scored as 0 = normal; 1 = minimal infiltration of inflammatory cells in periarticular tissue; 2 = mild infiltration; 3 = moderate infiltration, with moderate edema; 4 = marked infiltration, with marked edema; and 5 = severe infiltration, with severe edema. Cartilage damage and bone erosion were scored as 0 = normal; 1 = minimal (minimal-to-mild loss of cartilage and bone); 2 = mild (mild loss of cartilage and bone); 3 = moderate (moderate loss of cartilage and bone); 4 = marked (marked loss of cartilage and bone); and 5 = severe (severe diffuse loss of cartilage and bone). From each joint, 6 areas of 2 sections were used to provide a representative sample of the whole joint. Mean scores were the average of all section scores for each animal.

The synovial pH values were measured by inserting a 20 G needle pH electrode (IC-401, Specialty Sensors, LCC, Hamden, CT, USA) into the tibiotarsal joint.

### Assessment of arthritic pain

To assess mechanical nociceptive responses, animals were tested for withdrawal thresholds to mechanical stimuli (von Frey filaments, Touch-Test, North Coast Medical, Morgan Hill, CA) applied to the plantar aspect of the hindpaw. As described^44^, mice (n ≥ 6 per group) were pre-trained for 1 to 2 hr each day for 2 days before the test. Von Frey fibers were applied 5 times at 5-sec intervals to the plantar surface of each hindpaw at various times after injections. The paw withdrawal threshold (PWT) was determined when paw withdrawal was observed in more than 3 of 5 applications.

### Measurement of cytokine levels in serum

Before (0 weeks) or after (4, 8, 12 weeks) CFA injection, mice were sacrificed and blood was collected by cardiac puncture. For serum samples, blood was left to clot for 30 min at 4°C, followed by centrifugation for 20 min at 2000 × g. Serum was aliquot and stored at −80 °C. Serum level of tumor necrosis factor α (TNF-α) (#MTA00B) or interleukin 6 (IL-6) (#M6000B) was measured with ELISA kits from R&D system (Minnesota, USA) as instructed.

### Quantitative RT-PCR

Lumbar 1–5 (L1-5) DRG ipsilateral and contralateral to injected paws were removed at 0, 4, 8, or 12 weeks for RNA extraction, with DRG from 0 weeks as a control. RNA extraction was performed as described^45^. Each DRG pool contained at least 10 DRG from 3 mice. RNA was extracted by using the RNeasy kit (Qiagen, Valencia, CA). Each gene primer (100 nM), derived cDNA, and master mix (SYBR green I and AmpliTaq Gold DNA polymerase [Applied Biosystems, Foster City, CA]) were mixed for PCR reactions and product detection by using the ABI Prism 7300 system. For each assay, preparations were run in triplicate. The thermal cycling conditions were 95 °C for 10 min, followed by 40 cycles of 95 °C for 15 s, and 60 °C for 1 min. The threshold cycle (Ct) values for both targets and the internal reference (mGAPDH) were measured from the same samples, and the expression of the target genes relative to that of mGAPDH was calculated by the comparative Ct method.

The primer sequences were for TDAG8 (197 bp), 5′-atagtcagcgtcccagccaac (forward)/5′-cgcttcctttgcacaaggtg (reverse); ASIC3 (100 bp), 5′-tttcacctgtcttggctcct (forward)/5′-caggatagtggtggggattg (reverse); TRPV1 (151 bp), 5′-tctccactggtgttgagacg (forward)/5′-gggtctttgaactcgctgtc (reverse); and mGAPDH (233 bp), 5′-ggagccaaacgggtcatcatctc (forward)/ 5′-gaggggccatccacagtcttct (reverse).

### Statistical analysis

All data are presented as mean ± SEM. One-way or two-way ANOVA with post-hoc Bonferroni test was used to compare results for multiple groups. Nonparametric Mann-Whitney U test was used to compare results for histologic scores and cytokine analysis. P < 0.05 was considered statistically significant.

## Electronic supplementary material

Supplementary information
