# Supplementary material for: TDAG8, TRPV1, and ASIC3 involved in establishing hyperalgesic priming in experimental rheumatoid arthritis
Source: Sci Rep. 2017 Aug 21;7:8870. doi: 10.1038/s41598-017-09200-6 (PMC5566336; doi:10.1038/s41598-017-09200-6)
Supplement: Supplementary file 1 — Supplementary information [file 41598_2017_9200_MOESM1_ESM.pdf]

TDAG8, TRPV1, and ASIC3 involved in establishing hyperalgesic priming in experimental rheumatoid arthritis

Wei-Shan Hsieh[1,#], Chia-Chi Kung[1,2#], Shir-Ly Huang[3], Shih-Chang Lin[4] and Wei-Hsin Sun[1] *

Supplementary data:

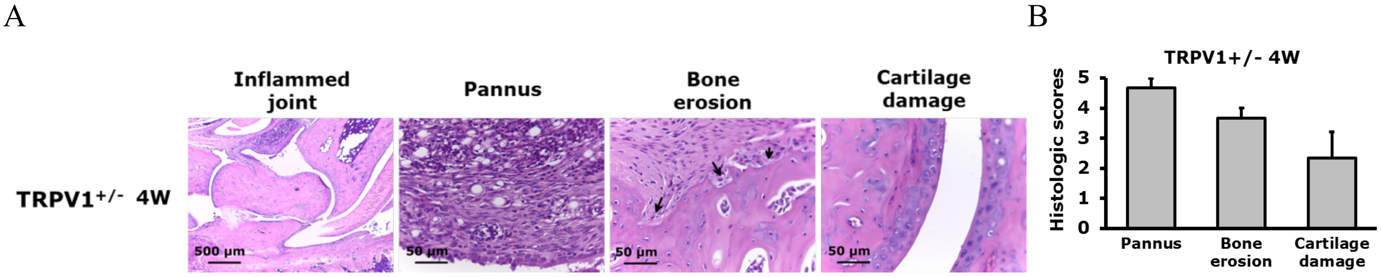

Suppl Fig. 1 **Histology of ipsilateral joint in arthritic TRPV1$^{+/-}$ mice.** Samples of ipsilateral TRPV1$^{+/-}$ joints at 4 week stained with hematoxylin and eosin. Data are mean±SEM severity score for synovial inflammation, bone erosion, and cartilage damage.

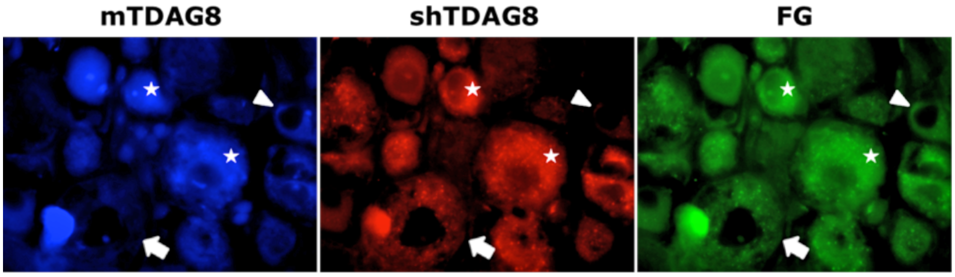

Suppl Fig. 2 **TDAG8 is expressed in joint afferents.** The mouse was intraplantarly injected with shTDAG8 (red fluorescence) and intra-articularly injected with fluorogold (FG, green fluoresence) for 7 days, and mouse was then intra-articularly injected with 5μg CFA. After 1 week, ipsilateral DRG was isolated, sectioned and stained with anti-TDAG8 antibody (blue fluorescence). * indicates neurons with FG-labeling (FG^{+}) and shTDAG8 expression (shTDAG8^{+}). Arrowheads indicate FG^{+}/ shTDAG8^{-} neurons express TDAG8 protein. Arrows indicate FG^{+}/ shTDAG8^{+} neurons do not express TDAG8 protein.

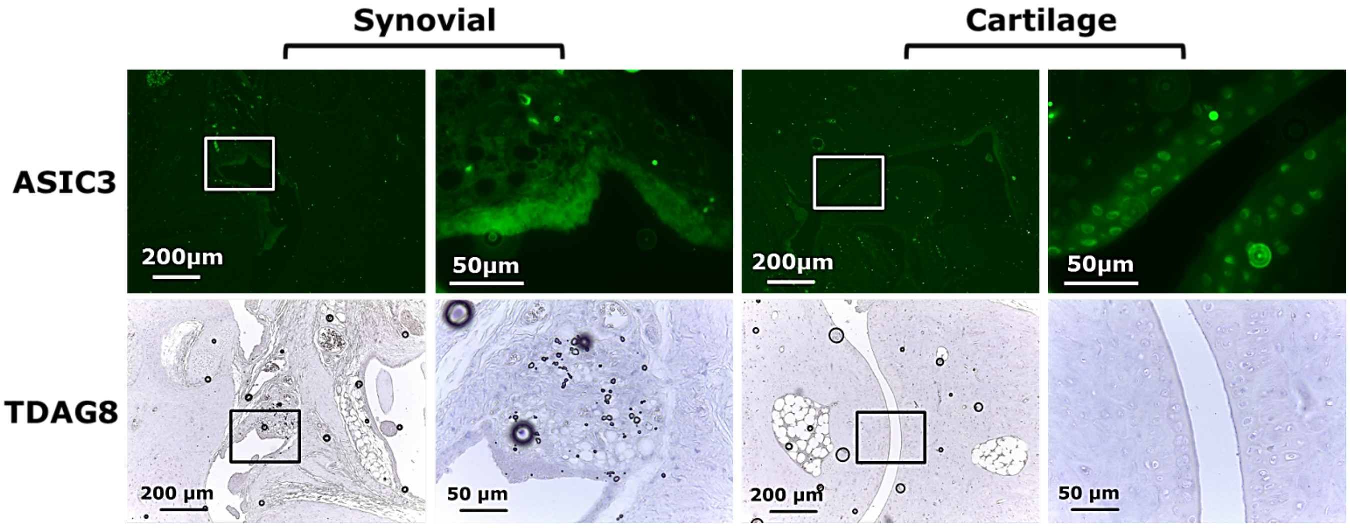

Suppl Fig. 3 **ASIC3 is expressed in synovial sublining and cartilage, but TDAG8 is expressed in synovial sublining.** Samples of joints from WT mice at 12 weeks were stained with anti-ASIC3 or anti-TDAG8 antibodies, followed by FITC- or AP-conjugated secondary antibodies, respectively. Synovial sublining and cartilage was showed. Green fluoresence indicates ASIC3-positive immunoreactivity. Blue dots indicate TDAG8-positive imunoreactivity.
